# Evaluation of *Saccharomyces cerevisiae* fermented Moutai distiller’s grains as an alternative ingredient for growing-finishing pigs

**DOI:** 10.1093/tas/txaf143

**Published:** 2025-10-24

**Authors:** Chunqi Lu, Xiaomeng Liu, Nanling Song, Guangshuang Tu, Youfeng Jiang, Qiuyue Fu, Huixin Xiong, Hang Yu, Si Gao, Yingjun Li, Shuai Wang

**Affiliations:** Kweichow Moutai (Group) Circular Economy Industrial Investment and Development Co., Ltd, Zunyi 563000, China; National Key Laboratory of Agricultural Microbiology, Wuhan 430070, China; Frontiers Science Center for Animal Breeding and Sustainable Production, College of Animal Science and Technology, Huazhong Agricultural University, Wuhan 430070, China; Kweichow Moutai (Group) Circular Economy Industrial Investment and Development Co., Ltd, Zunyi 563000, China; National Key Laboratory of Agricultural Microbiology, Wuhan 430070, China; Frontiers Science Center for Animal Breeding and Sustainable Production, College of Animal Science and Technology, Huazhong Agricultural University, Wuhan 430070, China; Kweichow Moutai (Group) Circular Economy Industrial Investment and Development Co., Ltd, Zunyi 563000, China; National Key Laboratory of Agricultural Microbiology, Wuhan 430070, China; Frontiers Science Center for Animal Breeding and Sustainable Production, College of Animal Science and Technology, Huazhong Agricultural University, Wuhan 430070, China; Kweichow Moutai (Group) Circular Economy Industrial Investment and Development Co., Ltd, Zunyi 563000, China; National Key Laboratory of Agricultural Microbiology, Wuhan 430070, China; Frontiers Science Center for Animal Breeding and Sustainable Production, College of Animal Science and Technology, Huazhong Agricultural University, Wuhan 430070, China; Frontiers Science Center for Animal Breeding and Sustainable Production, College of Animal Science and Technology, Huazhong Agricultural University, Wuhan 430070, China; National Key Laboratory of Agricultural Microbiology, Wuhan 430070, China; College of Life Science and Technology, Huazhong Agricultural University, Wuhan 430070, China; National Key Laboratory of Agricultural Microbiology, Wuhan 430070, China; Frontiers Science Center for Animal Breeding and Sustainable Production, College of Animal Science and Technology, Huazhong Agricultural University, Wuhan 430070, China

**Keywords:** carcass characteristics, fermented Moutai distiller’s grains, growing-finishing pigs, growth performance, intestinal barrier

## Abstract

Three experiments were conducted to evaluate the effects of incorporating *Saccharomyces cerevisiae* fermented Moutai distiller’s grains (**FMDG**) into diets for growing-finishing pigs across three body weight (**BW**) phases: 30 to 50 kg (Exp. 1), 50 to 75 kg (Exp. 2), and 90 to 130 kg (Exp. 3). The experimental diets were corn-soybean meal based with 0%, 5% or 10% FMDG. Metabolomic analysis revealed that fermentation enriched FMDG with beneficial compounds, including carbohydrates, quercetin, and tripeptides, compared to unfermented Moutai dried distiller’s grains. Results form the animal experiments showed that dietary inclusion of FMDG at levels up to 10% did not adversely affect growth performance in any phase. Notably, 5% FMDG inclusion during the later finishing phase (90 to 130 kg) significantly increased (*P *< 0.05) average daily feed intake (**ADFI**) and numerically improved average daily gain (**ADG**) and feed conversion ratio (**FCR**), reducing meat production cost by 0.54 RMB/kg. Apparent total tract digestibility (**ATTD**) of nutrients varied by phase, with enhanced crude protein and calcium utilization at 30 to 50 kg but reduced (*P *< 0.05) calcium digestibility at 90 to 130 kg. In later finishing pigs, inclusion of 5% and 10% FMDG significantly decreased (*P *< 0.05) serum endotoxin, diamine oxidase, and interleukin-6 levels. We further confirmed that dietary inclusion of FMDG increased (*P *< 0.05) the expression of tight junction proteins including claudin-4, occludin, and zonula occludens protein-1 (**ZO-1**) in the jejunum. Carcass traits and meat quality were maintained or improved, with increased (*P *< 0.05) loin eye area and meat redness (a*) in the 5% FMDG group. Overall, these findings demonstrate that FMDG can be effectively included at up to 10% in swine diets without compromising animal health and performance, with 5% FMDG providing particularly beneficial for later finishing pigs due to its positive impacts in improving feed efficiency, carcass traits and meat quality, as well as enhancing intestinal integrity.

## Teaser

Feedstuff deficiency, especially protein feed resources, poses a major constraint to sustainable livestock development. The current study offers comprehensive insights into the precise use of *Saccharomyces cerevisiae* fermented Moutai distiller’s grains as an alternative ingredient in growing-finishing pigs diets, with beneficial effects in improving feed efficiency, carcass traits and meat quality, as well as enhancing intestinal integrity.

## Introduction

The global livestock industry faces persistent pressure to identify sustainable and cost-effective alternatives to traditional feed ingredients, such as corn and soybean meal. Feedstuff deficiency, particularly protein feed resources, is a major constraint on livestock development in China. Agricultural byproducts have garnered significant interest for their potential to reduce feed costs and improve the circular economy in recent years (Sandström et al. 2022). Chinese Baijiu is one of the six major distilled spirits worldwide, with a considerable output. Distillers’ grains (**DG**) are organic solid residues of sorghum, corn and other grains in Chinese Baijiu production, with high nutritional value such as starch, protein and fat (Zhang et al. 2024). Annually, over 100 million tons of DG as waste are generated from the Chinese Baijiu production (Zhi et al. 2017). However, conventional waste management usually causes environmental pollutants and bioresource wasting. Feeding takes the dominant status in DG utilization, whereas the demand from the livestock industry may become restricted due to the nutritional variability and anti-nutritional factors including remained alcohol and mycotoxins (Murtaza et al. 2025). Through microbial fermentation, DG are valorized via bioconversion into high-value fermented protein feed, offering a promising solution to the critical shortage of protein supplements in the feed industry (Iram et al. 2020).

Moutai distiller’s grains (**MDG**) is a unique byproduct from the production of Kweichow Moutai liquor, which using sorghum and wheat as the raw materials. Unlike conventional distiller’s dried grains with solubles (**DDGS**), MDG retains a distinctive profile of fermentation metabolites from Moutai liquor production, including bioactive compounds (e.g. polyphenols and flavonoids) that confer health benefits to animals (Chen et al. 2023). In addition, microbial fermentation can considerably improve the nutritive value of DG, such as enriching proteins, probiotics, and microbial metabolites. *Saccharomyces cerevisiae* is widely used for DG fermentation to improve the suitability of the DG. Our recent findings revealed that inclusion of up to 10% Moutai dried distiller’s grains (**MDDG**) in broilers’ diet significantly decreased growth performance, whereas inclusion of fermented Moutai distiller’s grains (**FMDG**) up to 10% did not impair broilers performance, because of the nutrient ingredients of FMDG is superior to MDDG (Zheng et al. 2025). Cheng et al. (2022) demonstrated that adding 30% FMDG to the feed of finishing cattle exhibited no adverse effects on growth performance and meat quality. The recommended amount of Baijiu DG for weaned pigs (< 20 kg BW), growing pigs (20 to 50 kg BW), and growing-finishing pigs (50 to 100 kg BW) are 4%, 15% and 20% (Zhang et al. 2024). Due to the variations in brewing processes and raw materials, the nutritional composition of DG from different sources varies widely. Therefore, the optimum level of a new DG product in pig diets need to be determined before its application.

However, there is a scarcity of comprehensive studies evaluating the effect of FMDG inclusion in swine diets, particularly across different growth phases, on growth performance, nutrient utilization, and meat quality. This study compared the metabolomic profilings between FMDG and MDDG by metabolomic analysis. The objective of this study was to evaluate the potential of FMDG as an alternative ingredient for growing-finishing pigs through three sequential experiment (30 to 50 kg, 50 to 75 kg, and 90 to 130 kg).

## Materials and methods

The Institutional Animal Care and Use Committee at Huazhong Agricultural University reviewed and approved the protocols for three experiments (approval number: HZAUSW-2024-0063). The MDG samples were obtained from Kweichow Moutai Group in the Moutai town, Renhuai city of Guizhou Province, China. The production process for MDDG and FMDG has been previously described (Zhang et al. 2021; Zheng et al. 2025).

### Metabolomic analysis

Four MDDG and four FMDG samples were subjected to metabolomic analysis. Samples (50 ± 5 mg) were dissolved in 400 μL of a methanol: water (4:1, v/v) solution containing 0.02 mg/mL L-2-chlorophenylalanin as internal standard, and vortexed for 6 min, followed by ultrasound at 5 °C and 40 kHz for 30 min. The samples were then placed at −20 °C for 30 min, and were centrifuged at 4 °C and 13,000 g for 15 min. The supernatants were collected and filtered through 0.22 μm syringe filters for LC-MS/MS analysis. Metabolomics sequencing in this work was provided by Majorbio Biotech Co., Ltd The sequencing method of untargeted mass spectrometry was the same as in our previous study (Xue et al., 2022). Here, the significant differences in metabolites between MDDG and FMDG groups were identified with VIP value > 1 and *P* value < 0.05.

### Animals and experimental design

Samples of the FMDG has already been analyzed for dry matter (**DM**), crude protein (**CP**), ether extract, crude ash, crude fibre, calcium, phosphorus and amino acids in our previous study (Zheng et al. 2025). For these three experiments, the experimental diets were corn-soybean meal based with 0%, 5% or 10% FMDG (Table 1). The inclusion levels of FMDG were selected based on previous studies (Li et al. 2019; Huang et al. 2020). All diets were manufactured and animal experiments were conducted at the Guizhou Forryea Technology (Group) Co., Ltd.

**Table 1.** Composition and nutrient content of the experimental diets (as-fed basis)^1^.

Item	Experiment 1			Experiment 2			Experiment 3		
	FMDG, %			FMDG, %			FMDG, %		
	0	5	10	0	5	10	0	5	10
**Ingredients, %**									
**Corn**	53.87	53.87	53.87	58	58	58	61.67	63.68	59.71
**Wheat flour**	15	15	15	15	15	15	15	15	15
**Soybean meal**	12	12	12	9.48	9.48	9.48	4.85	4.9	3.4
**Wheat bran**	10	5	0	10	5	0	10	3	3
**FMDG**	0	5	10	0	5	10	0	5	10
**Rapeseed cake**	3	3	3	3	3	3	4	4	4
**Soybean oil**	2.2	2.2	2.2	1.3	1.3	1.3	1.15	0.95	1.5
**Limestone**	0.95	0.95	0.95	0.9	0.9	0.9	1	1	1
**Hydryoxyapetite 20%**	0.8	0.8	0.8	0.7	0.7	0.7	0.65	0.8	0.75
**Montmorillonite**	0.7	0.7	0.7						
**Lys 98%**	0.51	0.51	0.51						
**Lys 70%**				0.7	0.7	0.7	0.8	0.8	0.84
**NaCl**	0.3	0.3	0.3	0.35	0.35	0.35	0.3	0.3	0.2
**L-Thr 98.5%**	0.2	0.2	0.2	0.17	0.17	0.17	0.21	0.2	0.22
**Tannic acid**	0.08	0.08	0.08						
**DL-Met**	0.07	0.07	0.07	0.07	0.07	0.07	0.04	0.04	0.04
**L-Tyr 98.5%**	0.05	0.05	0.05	0.04	0.04	0.04	0.05	0.05	0.05
**Guanidineacetic acid**				0.05	0.05	0.05			
**Val**	0.03	0.03	0.03				0.04	0.04	0.05
**Premix^2^**	0.24	0.24	0.24	0.24	0.24	0.24	0.24	0.24	0.24
**Total**	100	100	100	100	100	100	100	100	100
**Price, RMB/t**	3073.11	3080.89	3088.6	2868.77	2878.29	2887.81	2852.14	2856.14	2860.14
**Calculated nutrional value**									
**NE, Kcal/kg**	2380.51	2366.51	2352.51	2572	2596	2619	2572	2596	2619
**Crude protein, %**	14.22	14.7	15.19	13.51	13.63	13.74	13.51	13.63	13.74
**Standardized ileal digestibility (SID) of Lys, %**	1.34	1.35	1.35	0.85	0.85	0.85	0.85	0.97	0.97
**Calcium, %**	0.65	0.68	0.7	0.6	0.64	0.63	0.6	0.64	0.63
**Phosphorus, %**	0.56	0.53	0.5	0.52	0.51	0.5	0.52	0.51	0.5

1 Experiment 1 was carried out from 30 to 50 kg, experimental 2 from 50 to 75 kg, and experimental 3 from 90 to 130 kg, respectively.

2 The premix provided the following per kg of feed: Vitamin A 5805 IU, Vitamin D_3_: 2025 IU, Vitamin E 16.2 mg, Vitamin K_3_ 1.35 mg, Vitamin B_1_ 1.485 mg, Vitamin B_2_ 5.4 mg, Vitamin B_6_ 2.7 mg, Vitamin B_12_ 0.01 mg, D-Biotin 0.14 mg, D-Pantothenic acid 12.56 mg, Folic acid 1.755 mg, Niacinamide 17.55 mg, Copper 5.2 mg, Iron 26 mg, Zinc 24 mg, Manganese 21.2 mg, Iodine 0.4 mg, Selenium 0.36 mg.

#### Experiment 1

A total of 544 crossbred (Duroc × Landrace × Yorkshire) growing pigs (30 ± 3.64 kg) were used in a 24-d trial with 41 to 47 pigs per pen and 4 replicate pens per treatment. Pens of pigs were assigned into treatments in a randomized complete block design and pigs were weighed at days 1 and 24, and feed intake of each pen were recorded during d 1 to 24 to determine average daily feed intake (**ADFI**), average daily gain (**ADG**), and feed conversion ratio (**FCR**). Three days prior to the conclusion of the experiment, fecal samples were collected from two pigs per pen to estimate apparent total tract digestibility (**ATTD**) of nutrients. Fecal samples were pooled by pen and were added with 10% hydrochloric acid, and then stored at −20 °C until analysis. On d 24, jugular vein blood samples were collected from two pigs in each pen using heparin-free vacuum blood tubes. Serum was separated to analyze biochemical parameters.

#### Experiment 2

A total of 510 growing pigs (Duroc × Landrace × Yorkshire, initial BW: 49.65 ± 2.86 kg) were used in a 25-d trial with 40 to 43 pigs per pen and 4 replicate pens per treatment. Pens of pigs were assigned to treatments in a completely randomized design with initial weight as the blocking factor. Pens of pigs were weighted at days 1 and 25, and feed intake were recorded during d 1 to 25 to determine ADFI, ADG, and FCR. The procedures of fecal and blood samples collection were the same as in Experiment 1.

#### Experiment 3

A total of 369 crossbred (Duroc × Landrace × Yorkshire) growing-finishing pigs (92.57 ± 4.47 kg) were used in a 44-d trial with 28 to 32 pigs per pen and 4 replicate pens per treatment. Pens of pigs were assigned into treatments in a randomized complete block design and pigs were weighed at days 1 and 44, and feed intake of each pen were recorded during d 1 to 44 to determine ADFI, ADG, and FCR. The procedures of fecal and blood samples collection were the same as in Experiment 1. At the end of the experiment, two pigs in each pen were randomly selected and slaughtered to evaluate carcass traits. Slaughter was conducted under commercial conditions at the Guizhou Qiansuxian Food Co., Ltd (Guiyang, China). Hot carcass weight was immediately recorded following slaughter, and dressing percentage was calculated based on hot carcass weight and live weight. The Carcass length was measured from the anterior edge of the symphysis pubis to the cranial edge of the first rib adjacent to the thoracic vertebra. The right carcasswas split and sectioned between 10th and 11th ribs for measurements of longissimus dorsi area, fat depth, and 24-h pH (pH-Star, DK2730, Herlev, Denmark). Drip loss was measured according to previously reported method (King et al. 2000). Loin muscle marbling was scored based on NPPC (1994) guidelines and meat colour parameters including lightness (L*), redness (a*), and yellowness (b*) were determined with a Chroma meter CR410 (Konica Minolta, Japan).

Fragments of jejunal tissue, liver, and spleen were collected and fixed in 4% paraformaldehyde for histomorphology. Additionally, samples from the same sites of jejunum were excised, flash-frozen in liquid nitrogen, and kept frozen at −80 °C until analysis.

### Chemical analyses

Fecal samples were thawed and dried in a 65 °C forced-air drying oven and finely ground with a Wiley mill (Thomas Scientific, Swedesboro, NJ). One subsample of each diet and feces was analyzed for CP, crude fat, ash, calcium, and phosphorus according to AOAC International (2007) procedures. Following chemical analysis, the ATTD of nutrients was calculated for each diet according to the formula adapted from She et al. (2018): ATTD (%) = [(Nutr_in—_Nutr_out_)/Nutr_in_] × 100, where Nutr_in—_Nutr_out_ are the nutrient intake in the diet DM and output in feces DM, respectively.

### Serum biochemical parameters

Serum biochemical parameters, including endotoxin, diamine oxidase, interleukin-6 (**IL-6**), and interleukin-10 (**IL-10**) were analyzed using the enzyme-linked immunosorbent assay (**ELISA**) method with commercial ELISA kits (MIBio, Shanghai, China). The activities of total superoxide dismutase (**T-SOD**) and catalase (**CAT**) in serum were determined with a colorimetric method by the specific assay kits according to the manufacturer’s protocols (Nanjing Jiancheng Bioengineering Institute, Nanjing, China).

### Histological analysis

The jejunal segments, liver and spleen tissues fixed in 4% paraformaldehyde from Experiment 3 were embedded in paraffin. The samples were sectioned at 5 μm and were stained with hematoxylin and eosin. Histomorphometry observation was using an Olympus BX53 microscope (Olympus, Tokyo, Japan). Villus height and crypt depth were measured at 4 × 10 magnification. At least 10 well-oriented intact villi and the associated crypt depth were measured for each pigs. Microscopic observations in liver and spleen were quantified as already described (Gerez et al. 2015).

### Western blot analysis

Relative protein levels for claudin-4, occludin, and zonula occludens protein-1 (**ZO-1**), obtained from the jejunal tissue, were determined by western blot technique as perviously described (Ma et al. 2015). Briefly, after extraction, proteins were separated on sodium dodecyl sulphate polyacrylamine geles and electrotransferred to PVDF membranes. The membranes were probed with primary antibodies at appropriate concentration. Rabbit polyclonal anti-β-actin (ABclonal, Wuhan, China) was used as control. After being washed with Tris-Tween-20 buffer (pH 7.4), membranes were incubated with secondary antibody (horseradish peroxidase-conjugated goat anti-rabbit IgG; Boster Biological Technology, Wuhan, China) at a 1:4,000 dilution for 1 h at room temperature. The chemifluorescenes intensity of specific bands was obtained with a Tanon-5200 Chemiluminescent Imaging System (Tanon, China). Protein expression levels were quantified by normalizing the band of interest to the β-actin loading control.

### Statistical analysis

For growth performance and ATTD data, the pen was treated as the experimental unit. For carcass traits, serum biochemical indices, jejunal morphology, and jejunal barrier function data, the growing-finishing pig was considered as the experimental unit. Data were analyzed as a completely randomized design by one-way ANOVA using the GLM procedure of SAS version 9.4 (SAS Inst. Inc., Gary, NC). All data were checked for normal distribution and homogeneous variance with the NUIVARIATE procedure. Data are shown as the Lsmeans and SEM. Statistical differences among treatments were determined using Student Newman Keuls Multiple Range Test. A *P*-value ≤ 0.01 was considered highly significant, a *P*-value ≤ 0.05 was considered significant, and a tendency for differences was declared between *P *> 0.05 and *P *≤ 0.10.

## Results

### Metabolite differences between MDDG and FMDG

**Fig. 1.**
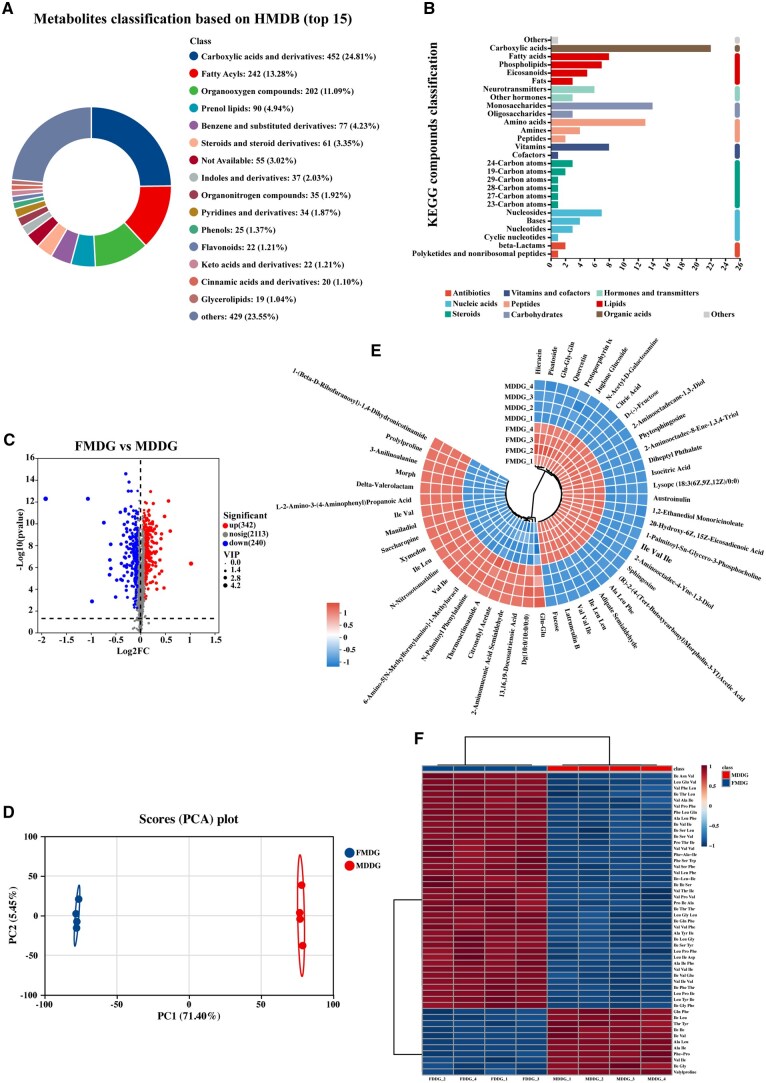
Comparison of metabolomic profilings between FMDG and MDDG. Metabolites classification based on a) HMDG and b) KEGG database. c) Volcano plot and d) PCA analysis of metabolites in FMDG and MDDG. e) Circular heatmap of cluster analysis of the top 50 metabolites with *P *< 0.05 calculated by *T*-test. (F) Heatmap of cluster analysis of the top 50 short peptides with *P *< 0.05 calculated by *T*-test.

Metabolomic analysis was performed to reveal the metabolite differences between MDDG and FMDG (Fig. 1). Mass spectrometry data analysis identified a total of 2,695 metabolites, including 1,822 known compounds mapped onto Human Metabolome Database (**HMDB**) and 873 known compounds mapped onto Kyoto Encyclopedia of Genes and Genomes (**KEGG**) database. Among the metabolites classification based on HMDB, predominant ones were in the order of carboxylic acids and derivatives, fatty acyls, organooxygen compounds, prenol lipids, benzene and substituted derivatives, steroids and steroid derivatives (Fig. 1a). Among the 873 metabolites mapped onto KEGG database, predominant ones were in the order of carboxylic acids, monosaccharides, amino acids, vitamins, and fatty acids (Fig. 1b). We found 582 differential metabolites (Table S1), of which 342 were upregulated and 240 were downregulated in FMDG as compared with MDDG (Fig. 1c). In addition, principal component analysis (**PCA**) analysis was performed to obtain a high level of group separation (Fig. 1d). Circular heatmap showing the abundance of the top 50 metabolites having VIP score > 1 is shown in Fig. 1e. FMDG was found to have increased levels of carbohydrates and carbohydrate conjugates, quercetin, and tripeptide. We further analyzed the short peptides abundance and found FMDG had higher tripeptide but lower dipeptide levels than MDDG (Fig. 1f).

### Growth performance

The growth performance data of growing-finishing pigs at different stages fed diets containing no co-product or diets with 5% or 10% FMDG is shown in Table 2. For Exp. 1 (30 to 50 kg phase), no significant differences (*P *> 0.05) were observed between the dietary treatments for any growth performance parameter. Initial and final body weights, ADFI, ADG, and FCR were statistically similar across all groups. However, the meat production cost was increased by 0.17 and 0.30 RMB/kg in pigs fed diets containing 5% or 10% FMDG, respectively.

**Table 2.** Effects of increasing FMDG inclusion on growth performance of growing-finishing pigs at different stages^1^.

Item	Ctrl	FMDG, %		SEM	*P*-value
		5	10		
**30 to 50 kg (Exp. 1)**					
** Initial body weight, kg**	30.79	31.2	31.07	1.05	0.989
** Final body weight, kg**	49.16	49.14	48.06	1.23	0.931
** ADFI, kg/d**	1.69	1.67	1.62	0.04	0.763
** ADG, g/d**	734	705	680	13	0.232
** FCR**	2.3	2.31	2.38	0.04	0.765
** Mortality, %**	0	0.56	0	-	-
** Meat cost, RMB/kg**	7.07	7.24	7.37	-	-
**50 to 75 kg (Exp. 2)**					
** Initial body weight, kg**	49.63	49.65	49.65	1.24	1
** Final body weight, kg**	75.38	75.58	74.28	1.23	0.941
** ADFI, kg/d**	2.38	2.43	2.4	0.04	0.686
** ADG, g/d**	1002.4	1035.32	986.63	14.89	0.446
** FCR**	2.37	2.35	2.43	0.03	0.186
** Mortality, %**	1.16	0	0	-	-
** Meat cost, RMB/kg**	6.81	6.76	7.03	-	-
**90 to 130 kg (Exp. 3)**					
** Initial body weight, kg**	92.42	92.1	93.2	1.29	0.948
** Final body weight, kg**	130.7	134.36	130.39	1.36	0.455
** ADFI, kg/d**	3.02^b^	3.16^a^	3.19^a^	0.28	0.013
** ADG, g/d**	884	966	848	29	0.258
** FCR**	3.42	3.3	3.8	0.12	0.217
** Mortality, %**	3.15	1.72	1.59	-	-
** Meat cost, RMB/kg**	9.88	9.34	10.72	-	-

a ,

b Superscripts represent significant differences (*P *< 0.05).

1 Data are the means of four replicates with 41 to 47 growing-finishing pigs per pen (Exp.1), 40 to 43 growing-finishing pigs per pen (Exp. 2), 28 to 34 finishing pigs per pen (Exp. 3) for performance data, respectively.

Similar to Exp. 1, there were no statistically significant differences (*P *> 0.05) in growth performance metrics, including ADFI, ADG, and FCR, among the treatment groups in Exp. 2 (50 to 75 kg phase). Compared to the control, dietary inclusion of 5% or 10% FMDG decreased the mortality. Notably, pigs in the 5% FMDG group achieved the lowest FCR numerically and consequently had the lowest meat cost.

For Exp. 3 (90 to 130 kg phase), pigs in both 5% and 10% FMDG dietary treatments had higher (*P *< 0.05) ADFI than the control. The 5% FMDG group showed a substantially higher numerical ADG compared to the control and the 10% FMDG groups, although the difference was not statistically significant (*P *= 0.258). The 5% FMDG group also had the lowest numerical FCR. Dietary inclusion of 5% or 10% FMDG decreased the mortality. In addition, the meat production cost was decreased by 0.54 RMB/kg in pigs fed diets containing 5% FMDG.

### ATTD of nutrients in diets

Values for the ATTD of nutrients are presented in Table 3. For Exp. 1 (30 to 50 kg phase), dietary inclusion of 10% FMDG tended (*P *= 0.057) to increase the ATTD of crude protein compared to the control. The 10% FMDG group had higher (*P *< 0.05) ash digestibility than both the control and 5% FMDG groups. Compared with the control, dietary inclusion of FMDG at 5% and 10% increased (*P *< 0.05) the ATTD of calcium. No significant differences (*P *> 0.05) were observed between the dietary treatments for ATTD of crude fat and phosphorus.

**Table 3.** Effects of increasing FMDG inclusion on apparent total tract digestibility of nutrients of growing-finishing pigs at different stages^1^.

Item	Ctrl	FMDG, %		SEM	*P*-value
		5	10		
**30 to 50 kg (Exp. 1)**					
** Crude protein, %**	82.99	84.57	86.1	0.56	0.057
** Crude fat, %**	84.14	86.27	87.42	0.71	0.159
** Ash, %**	37.34^b^	47.62^b^	57.45^a^	3.15	0.013
** Calcium, %**	68.16^b^	74.92^a^	79.21^a^	1.56	0.001
** Phosphorus, %**	77.78	79.91	82.64	1.1	0.206
**50 to 75 kg (Exp. 2)**					
** Crude protein, %**	82.49^a^	80.77^a^ ^b^	75.60^b^	1.24	0.041
** Crude fat, %**	81.74^b^	82.48^b^	88.04^a^	1.21	0.048
** Ash, %**	31.89	23.84	23.84	1.82	0.095
** Calcium, %**	53.93	49.81	52.65	1.91	0.708
** Phosphorus, %**	65.15	58.5	67.12	2.68	0.427
**90 to 130 kg (Exp. 3)**					
** Crude protein, %**	80.59	76.05	71.68	1.92	0.169
** Crude fat, %**	86.37	77.01	78.42	1.85	0.067
** Ash, %**	41.15	38.20	48.36	1.98	0.084
** Calcium, %**	52.75^a^	43.16^b^	31.56^c^	2.92	0.001
** Phosphorus, %**	48.39	47.8	47.56	1.71	0.983

a -.

c Superscripts represent significant differences (*P *< 0.05).

1 Data are the means of four replicates per treatment.

For Exp. 2 (50 to 75 kg phase), the 10% FMDG group had lower (*P *< 0.05) ATTD of crude protein compared to the control. Conversely, the ATTD of crude fat was higher (*P *< 0.05) in pigs fed diet containing 10% FMDG compared to the control and 5% FMDG groups. No significant differences were observed for ash, calcium, or phosphorus digestibility in this phase.

For Exp. 3 (90 to 130 kg phase), the ATTD of calcium decreased (*P *< 0.05) dramatically with increasing FMDG inclusion. Dietary inclusion of 5% FMDG tended (*P *= 0.067) to decrease the ATTD of crude fat. The ATTD of ash tended (*P *= 0.084) to increase in pigs fed diet containing 10% FMDG compared to control. In addition, there were no statistically significant differences (*P *> 0.05) in ATTD of crude protein or phosphorus.

### Serum biochemical indices

The impact of dietary inclusion of FMDG on serum biochemical indices was highly dependent the growth phase (Table 4). There were no statistically significant differences (*P *> 0.05) in serum biochemical indices, including endotoxin, diamine oxidase, IL-6, IL-10, T-SOD, and CAT, among the treatment groups in Exp.1 (30 to 50 kg phase) and Exp. 2 (50 to 75 kg phase). However, for Exp. 3 (90 to 130 kg phase), dietary inclusion of 5% and 10% FMDG significantly decreased (*P *< 0.05) the serum endotoxin and diamine oxidase levels compared to the control. In addition, the pro-inflammatory cytokine IL-6 was significantly lower (*P *< 0.001) in pigs fed both inclusion levels of FMDG diets than that in the control animals. No significant differences (*P *> 0.05) were found for the antioxidant enzymes (T-SOD, CAT) or the anti-inflammatory cytokine IL-10, although IL-10 levels were numerically lower in the FMDG groups.

**Table 4.** Effects of increasing FMDG inclusion on serum biochemical indices of growing-finishing pigs at different stages^1^.

Item	Ctrl	FMDG, %		SEM	*P*-value
		5	10		
**30 to 50 kg (Exp. 1)**					
** Endotoxin, EU/mL**	7.16	6.27	6.74	0.35	0.305
** Diamine oxidase, pg/mL**	215.64	209.58	223.68	0.23	0.791
** IL-6, pg/mL**	557.76	482.34	537.81	8.11	0.583
** IL-10, pg/mL**	120.7	123.83	122.25	2.35	0.873
** T-SOD, U/mL**	408.68	404.92	330.2	17.08	0.101
** CAT, U/mL**	5.48	5.64	6.18	0.26	0.54
**50 to 75 kg (Exp. 2)**					
** Endotoxin, EU/mL**	7.74	7.31	8	0.22	0.439
** Diamine oxidase, pg/mL**	235.24	226.71	252.4	5.36	0.137
** IL-6, pg/mL**	586.81	607.01	532.79	35.85	0.701
** IL-10, pg/mL**	114.14	114.76	105.38	2.02	0.101
** T-SOD, U/mL**	397.02	336.85	393.86	20.99	0.439
** CAT, U/mL**	4.9	5.39	4.15	21.13	0.439
**90 to 130 kg (Exp. 3)**					
** Endotoxin, EU/mL**	6.09^a^	4.77^c^	5.17^b^	0.13	<0.001
** Diamine oxidase, pg/mL**	120.55^a^	105.39^b^	102.53^b^	2.2	<0.001
** IL-6, pg/mL**	540.11^a^	436.68^b^	450.44^b^	10.88	<0.001
** IL-10, pg/mL**	98.11	86.31	82.77	3.47	0.17
** T-SOD, U/mL**	63.18	69.06	66.13	2.01	0.142
** CAT, U/mL**	9.5	10.2	10.32	0.44	0.738

a -

c Superscripts represent significant differences (*P *< 0.05).

1 Data are the means of 8 growing-finishing pigs per treatment.

### Carcass traits and meat quality

Carcass traits and meat quality of finishing pigs from Exp. 3 are shown in Table 5. No significant differences (*P *> 0.05) were observed for dressing percentage, carcass diagonal length, or backfat thickness among the three dietary groups. Pigs in the 5% FMDG group had larger (*P *< 0.05) loin eye muscle area compared to both the control group and the 10% FMDG group. Dietary FMDG inclusion had no significant (*P *> 0.05) effect on marbling core, drip loss percentage, pH _24 h_, and yellowness (b*). The lightness (L*) was higher in pigs fed diet containing 5% FMDG, and dietary inclusion of 5% or 10% FMDG tended (*P *= 0.065) to increase the redness (a*) compared to control.

**Table 5.** Effects of increasing FMDG inclusion on carcass traits of finishing pigs (exp. 3)^1^.

Item	Ctrl	FMDG, %		SEM	*P*-value
		5	10		
**Carcass traits**					
** Dressing percentage, %**	75.66	76.36	77.1	0.38	0.326
** Carcass diagonal length, cm**	85.25	86.5	87	0.62	0.512
** Loin eye muscle area, cm**	36.18^b^	43.6^a^	36.29^b^	1.29	0.019
** Backfat thickness, mm**	25.09	28.63	27.79	1.26	0.510
**Longissimus muscle quality**					
** Marbling score**	2.13	2.5	2.88	0.15	0.113
** Drip loss, %**	2.9	2.55	2.82	0.17	0.701
** pH**	6.54	6.5	6.46	0.04	0.679
L* (lightness)	43.9^b^	46.5^a^	43.36^b^	0.46	0.006
a* (redness)	7.91	8.22	9.79	0.36	0.065
b* (yellowness)	3.89	3.97	4.23	0.17	0.710

a ,

b Superscripts represent significant differences (*P *< 0.05).

1 Data are the means of 8 growing-finishing pigs per treatment.

### Histological morphology

**Fig. 2.**
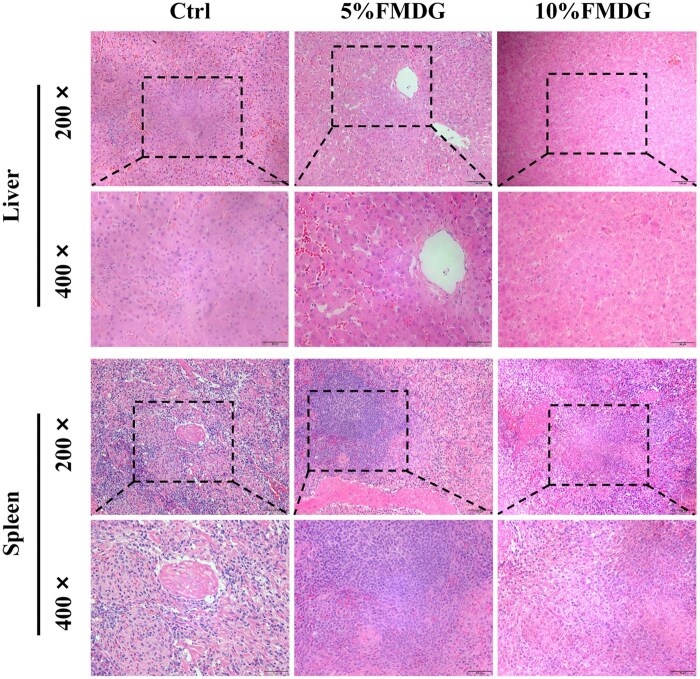
Representative photomicrographs of the liver and spleen morphology of finishing pigs (exp. 3) fed increasing diet inclusion rate of FMDG.

As shown in Table 6, dietary inclusion of FMDG at 5% and 10% levels did not produce any statistically significant effects on the jejunal morphology parameters, including villus height, crypt depth, and villus height to crypt depth ratio, compare to the control. The histological morphology of liver and spleen is presented in Fig. 2. No significant pathological changes were observed in the liver and spleen after the dietary inclusion of FMDG.

**Table 6.** Effects of increasing FMDG inclusion on jejunal morphology of finishing pigs (exp. 3)^1^.

Item	Ctrl	FMDG, %		SEM	*P*-value
		5	10		
**Villus height, μm**	437.13	463.13	447.63	14.39	0.775
**Crypt depth, μm**	249.87	246.75	247.63	8.50	0.989
**Villus height/crypt depth**	1.76	1.89	1.83	0.04	0.467

1 Data are the means of 8 growing-finishing pigs per treatment.

### Protein expression of jejunal tight junctions

**Fig. 3.**
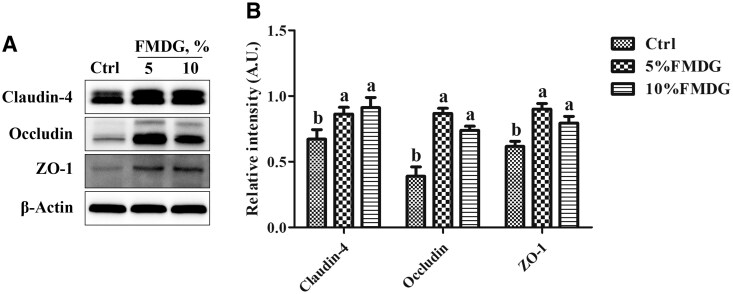
Inclusion of FMDG improved jejunal tight junction of finishing pigs (exp. 3). a) Western blot analysis of claudin-4, occludin, and ZO-1 expression in the jejunum of finishing pigs. b) Band intensity quantification of claudin-4, occludin, and ZO-1. β-actin was used as a protein loading control. Data are shown as mean ± SEM of 6 animals. Graphic bars without a common letter differ, *P *< 0.05.

The protein abundances of tight junctions in jejunum were presented in Fig. 3. Dietary inclusion of 5% and 10% FMDG significantly increased (*P *< 0.05) the jejunal claudin-4, occludin, and ZO-1 protein expression levels compared to the control.

## Discussion

Our previous work revealed that inclusion of 10% MDDG extremely decreased broiler growth performance, whereas inclusion of up to 10% FMDG did not (Zheng et al. 2025). This notable finding promotes us to further investigate the nutritional component differences between MDDG and FMDG by using non-targeted metabolomics. In the current study, metabolomic analysis indicated a substantial shift in the metabolite profile following fermentation, with an increase beneficial compounds, such as carbohydrates, quercetin, and tripeptides. These changes likely contribute to the improved nutritional quality and biological activity of FMDG. The elevated level of fucose in FMDG was closely associated with *S. cerevisiae* fermentation (Liu et al. 2018). Quercetin, a polyphenolic compound, was found to action as antioxidants to protect animal cells from radical oxygen or nitrogen injury (Raheem et al. 2025). *S. cerevisiae* secretes extracellular proteases that facilitate the hydrolysis of macromolecular proteins into tripeptides. Tripeptides possess anti-inflammatory and antioxidant properties, contributing to overall health of the host (Oliveira et al. 2022).

The present study demonstrated that dietary inclusion of FMDG at levels up to 10% had no adverse effects on growth performance of growing-finishing pigs across three distinct BW stages, which aligns with previous studies in cattle and broilers (Cheng et al. 2022; Zheng et al. 2025). Similarly, Li et al. (2019) observed that feeding diets containing 5%, 10% or 15% fermented Mao-tai lees showed no significant effects on growing-finishing pigs (40 to 110 kg BW). It is noteworthy that in the late two growing-finishing phases (50 to 75 kg BW, 90 to 130 kg BW), pigs fed diet containing 5% FMDG exhibited a numerical improvement in ADG and the lowest FCR, resulting in a reduction in meat production cost. These findings suggest that moderate inclusion of FMDG may improve feed efficiency in heavier pigs, possibly due to better adaptation of the gastrointestinal microbiota (Li et al. 2019; Zhang et al. 2025).

The results of ATTD analysis in the present study revealed a complex, and growth-phase dependent impact of FMDG on nutrients utilization in pigs. The tendency for improvement in crude protein digestibility and the significant enhancement of ash and calcium digestibility with FMDG inclusion during the early growth phase (30 to 50 kg BW) are likely a direct benefit of the fermentation process. Huang et al. (2003) also found that microbial fermented distiller’s grains improved ileal apparent digestibility of crude protein in growing pigs. However, in the 50 to 75 kg phase, a significant reduction in crude protein digestibility was observed with 10% FMDG inclusion, despite a concurrent increase in fat digestibility. The high crude fibre content in FMDG, while partially degraded by fermentation, may still increase the passage rate of chyme through the digestive tract, reducing the time available for proteolytic enzymes to act thus impairing protein digestion (Jha et al. 2015). Conversely, the enhanced fat digestibility could be attributed to fermentation-induced changes in the fiber matrix, which might better emulsify fat, or to the action of microbial lipases produced during the fermentation process (Kumar and Kanwar 2012). One striking result was the dramatic and dose-dependent decrease in calcium digestibility in the finishing phase (90 to 130 kg). A similar negative effect was also demonstrated in broilers fed a 10% FMDG diet (Zheng et al. 2025).

The analysis of serum biochemical indices reveals a highly growth-phase dependent effect of dietary FMDG inclusion on systemic health markers in pigs, with the most pronounced benefits observed during the finishing phase (90 to 130 kg BW). Besides growth phase, this phenomenon is likely attributable to the duration of feeding, as FDMG was administered for the longest period during the 90 to 130 kg phase. The significant reductions in serum endotoxin and diamine oxidase levels in the finishing phase pigs fed FMDG-containing diets provide compelling evidence of improved intestinal barrier function. Endotoxin is a component of gram-negative bacterial cell walls, and its increasing activity reflects increased intestinal permeability and intestinal barrier dysfunction (Vincenzo et al. 2024). Diamine oxidase is continuously released from the intestinal mucosa and its serum level reflects intestinal integrity damage (Wollin et al. 1998). The simultaneous reduction of both markers strongly suggests that FMDG inclusion at both 5% and 10% levels effectively enhanced intestinal barrier function in finishing pigs. We further confirmed that dietary inclusion of FMDG increased the expression of tight junction proteins including claudin-4, occludin, and ZO-1 in the jejunum, which might be attributed to the presence of polyphenols and fermentation-derived metabolites in FMDG. The significantly lower levels of the pro-inflammatory cytokine IL-6 in FMDG-fed groups further support the anti-inflammatory benefits of FMDG supplementation. The anti-inflammatory effects may be attributed to the polyphenolic compounds in FMDG, such as quercetin, which have been demonstrated antioxidant and anti-inflammatory properties (Lesjak et al. 2018). In addition, the metabolomic analysis revealed that fermentation significantly enriched FMDG with specific bioactive compounds, including tripeptides. Specific tripeptides containing Phe and Tyr, can effectively serve as scavengers of free radicals (Ye et al. 2022). Therefore, we postulate that the improvement in intestinal barrier function is not attribute to a single compound but is likely the results of a synergistic action of the fermented product in FMDG.

The architecture of intestinal mucosa is a well-known measure of gut health. Previous studies revealed that increasing inclusion levels of corn distillers dried grains with solubles (DDGS) in diets has a detrimental effect on intestinal morphology of pigs. Chen et al. (2025) found that inclusion of 20% DDGS resulted in a reduced villus height/crypt depth ratio in the jejunum and ileum of growing pigs. In the present study, the jejunal mucosa in finishing pigs fed the FMDG dietary treatments showed well-developed and parallel villi. No significant changes in jejunal morphology parameters were observed following dietary inclusion of FMDG at both 5% and 10% levels, suggesting FMDG does not elicit detrimental effects on intestinal development and integrity. The absence of significant changes in jejunal morphology, despite the marked improvements in tight junction proteins expression and systemic markers of intestinal integrity, suggests that the enhanced intestinal barrier function by FMDG was achieved without inducing structural hypertrophy or hyperplasia. This result indicates that FMDG inclusion improves gut health primarily through molecular and functional enhancements rather than structure remodeling, which is a favorable outcome for maintaining intestinal homeostasis. Furthermore, no noticeable alterations in the liver and spleen histology across all treatment groups provide compelling evidence for the safety of FMDG inclusion in swine diets.

The absence of significant differences in dressing percentage, carcass diagonal length, and backfat thickness among dietary treatments indicates that FMDG inclusion at both 5% and 10% levels does not compromise these fundamental carcass quality metrics. The majority of studies also showed no significant effects of feeding DDGS on carcass characteristics of growing-finishing pigs (Stein and Shurson 2009). Results of the current study are consistent with those findings for pigs fed DDGS. The loin eye muscle area serves as a reliable indicator of muscle development and carcass lean meat yield due to its strong correlation with carcass weight (Bu et al. 2021). In the present study, pigs fed 5% FMDG had larger loin eye muscle area. Another interesting finding of the present study is that loin muscle lightness (L*) and redness (a*) values were increased by inclusion of 5% or 10% FMDG, suggesting the meat colour was improved by FMDG. These results suggest that FMDG may offer advantages over conventional DDGS in terms of meat quality. Plant derived polyphenols can modulate meat colour by regulating muscle metabolism, and thereby improving meat quality (Wu et al. 2024). Moutai distiller’s grains are rich in polyphenols (Guo et al. 2025). The beneficial effects on meat quality are likely to be attributed to the presence of various bioactive compounds in FMDG, including flavonoids, phenolic acids, and alkaloids, as well as fermentation-derived metabolites (Falowo et al. 2014).

In conclusion, our data demonstrate that FMDG can be successfully incorporated into growing-finishing pig diets as a sustainable alternative protein source without compromising animal health and performance. Our findings reveal that dietary inclusion of FMDG at levels up to 10% had no adverse effects on growth performance across three distinct BW stages, with 5% FMDG inclusion providing particularly beneficial in the later finishing phase by improving feed efficiency, which reduced meat production cost by 0.54 RMB/kg. Additionally, inclusion of 5% FMDG in diet improved carcass traits and meat quality, as well as fortified intestinal barrier function of pigs, contributing to more efficient and environmentally friendly pork production.

## Supplementary Material

txaf143_Supplementary_Data

## Abbreviations

ADFI: average daily feed intake
ADG: average daily gain
ATTD: apparent total tract digestibility
BW: body weight
CAT: catalase
CP: crude protein
DDGS: distillers dried grains with solubles
DG: Distillers’ grains
DM: dry matter
ELISA: enzyme-linked immunosorbent assay
FCR: feed conversion ratio
FMDG: fermented Moutai distiller’s grains
HMDB: Human Metabolome Database
IL-6: interleukin-6
IL-10: interleukin-10
KEGG: Kyoto Encyclopedia of Genes and Genomes
MDDG: Moutai dried distiller’s grains
MDG: Moutai distiller’s grains
PCA: principal component analysis
SID: standardized ileal digestibility
T-SOD: total superoxide dismutase
ZO-1: zonula occludens protein-1
